# Caloric Restriction: A Novel Conditioning Strategy to Improve the Survival of Ischemically Challenged Musculocutaneous Random Pattern Flaps

**DOI:** 10.3390/nu15184076

**Published:** 2023-09-20

**Authors:** Andrea Weinzierl, Maximilian Coerper, Yves Harder, Michael D. Menger, Matthias W. Laschke

**Affiliations:** 1 Institute for Clinical and Experimental Surgery, Saarland University, 66421 Homburg, Germany; 2 Department of Plastic Surgery and Hand Surgery, University Hospital Zurich, 8091 Zurich, Switzerland; 3 Department of Plastic, Reconstructive and Aesthetic Surgery, Ospedale Regionale di Lugano, Ente Ospedaliero Cantonale (EOC), 6900 Lugano, Switzerland; 4 Faculty of Biomedical Sciences, Università della Svizzera Italiana, 6900 Lugano, Switzerland

**Keywords:** dietary restriction, caloric restriction, random pattern flap, necrosis, angiogenesis, nutrition, microcirculation, dorsal skinfold chamber, intravital fluorescence microscopy

## Abstract

Caloric restriction (CR) is a cost-effective and easy-to-perform approach to counteracting surgical stress. The present study therefore evaluates the tissue-protective effects of a 30% CR in musculocutaneous flaps undergoing ischemia. For this purpose, a well-established murine dorsal skinfold chamber model, in combination with random pattern musculocutaneous flaps, was used. C57BL/6N mice were divided at random into a CR group (n = 8) and a control group with unrestricted access to standard chow (n = 8). The CR animals were subjected to a 30% reduction in caloric intake for 10 days before flap elevation. Intravital fluorescence microscopy was carried out on days 1, 3, 5, 7 and 10 after flap elevation to assess the nutritive blood perfusion, angiogenesis and flap necrosis. Subsequently, the flap tissue was harvested for additional histological and immunohistochemical analyses. The CR-treated animals exhibited a significantly higher functional capillary density and more newly formed microvessels within the flap tissue when compared to the controls; this was associated with a significantly higher flap survival rate. Immunohistochemical analyses showed a decreased invasion of myeloperoxidase-positive neutrophilic granulocytes into the flap tissue of the CR-treated mice. Moreover, the detection of cleaved caspase-3 revealed fewer cells undergoing apoptosis in the transition zone between the vital and necrotic tissue in the flaps of the CR-treated mice. These results demonstrate that a CR of 30% effectively prevents flap necrosis by maintaining microperfusion on a capillary level and inhibiting inflammation under ischemic stress. Hence, CR represents a promising novel conditioning strategy for improving the survival of musculocutaneous flaps with random pattern perfusion.

## 1. Introduction

Caloric restriction (CR) is a form of dietary restriction (DR), which is defined as a reduction in caloric intake over a given period of time while maintaining adequate levels of macro- and micro-nutrients [1]. In contrast to starvation, it is sustainable over long time periods [2]. Long-term CR has been researched extensively for its ability to promote healthy aging and extend the organism’s life span [3]. It has been shown to reduce oxidative stress within various tissues and to attenuate certain metabolic and hormonal processes and mechanisms related to diseases, such as type II diabetes [4,5,6,7].

Previous studies show that DR is also able to counteract pathologies without a direct connection to food consumption and dietary habits, such as inflammatory diseases or chronic pain [8,9]. Initially, the described beneficial effects were believed to be irrelevant for clinical routines due to low compliance rates regarding long-term interventions [4,5,6]. However, more recent studies have shown that short-term dietary interventions may also increase resistance of tissues to multiple forms of acute stress [10,11]. For instance, 2 weeks of 30% CR prior to the induction of renal ischemia significantly improved the overall survival and kidney function in a murine model [11]. Similar effects have been demonstrated in hepatic, myocardial and cerebral tissue [12,13,14,15]. DR also attenuates insulin resistance and upregulates antioxidant activity [16].

Surgical interventions represent a form of critical tissue stress, as they disrupt nutritive tissue perfusion and cause a local inflammatory reaction. This can result in ischemic tissue damage, and eventually tissue necrosis. In plastic surgery, reconstructive procedures using tissue flaps are particularly prone to such ischemic tissue damage as large surface areas are undermined to transpose healthy tissue into a given tissue defect. Thus, ischemia-induced complications occur with rates cited as high as 37.5% [17]. Depending on the type of flap, blood perfusion is either supplied by a designated vascular axis that constitutes the vascular pedicle of a flap or through the musculocutaneous arterioles and/or the dermal vascular plexus passing through the flap’s base. In this case, the flap is considered a random pattern flap [18]. The changed arterial inflow may provide insufficient tissue perfusion and leave flap territories distant to the flap’s base undersupplied, as may also be the case in extended areas of axially perfused pedicled or microvascular flaps. The occurrence of hypoxia can potentially result in cellular dysfunction and ischemic cell death. On a macroscopic level, the ischemic stress then manifests as wound breakdown and partial or total flap necrosis. The treatment of these complications spans from prolonged local wound management to revision surgery, and is associated with a higher patient morbidity, as well as additional costs. Thus, there is a need for strategies to prevent ischemia-induced tissue complications in flap surgery. Strategies including local heat [19], local mechanical stress [20] or the administration of pharmacological substances [21] have been evaluated; however, they all present drawbacks, such as unwanted side effects or the need for additional equipment.

DR has been suggested as an attractive preoperative conditioning strategy to counteract surgical stress [22,23,24]. In contrast to other conditioning strategies, DR has almost no contraindications, except for malnourished patients and patients prone to hypoglycemia [25]. Moreover, it is not dependent on additional specialized equipment or pharmacological compounds. Beneficial effects of DR could also be detected in aged and overweight mice, indicating that even frail patients may benefit from preoperative DR [26]. In fact, CR has been studied in elderly and severely ill human patients with promising results [27,28], and thus may represent an opportunity for successfully implementing short-term DR in clinical routines. 

Based on these findings, the present study evaluates the effects of a 10-day 30% reduction in caloric intake onto random pattern musculocutaneous flaps in mice subjected to acute persistent ischemia. For this purpose, we used a well-established modified dorsal skinfold chamber model, which allows for the repeated analysis of nutritive flap perfusion and vascularization by means of intravital fluorescence microscopy.

## 2. Materials and Methods

### 2.1. Animals

All animal experiments in this study were carried out with the permission of the local governmental animal protection committee (Office for Consumer Protection, Saarbrücken, Germany; permit number: 10/2020). They were carried out in accordance with the European legislation on the protection of animals (Directive 2010/63/EU) and the NIH Guidelines on the Care and Use of Laboratory Animals (NIH publication #85-23 Rev. 1985).

The present study included 16 male C57BL/6N wild-type mice (Institute for Clinical and Experimental Surgery, Saarland University, Homburg, Germany). The animals were 12–24 weeks old and were only used if their body weight exceeded 26 g. One animal per cage was housed for the duration of the experiments to avoid any damage to the chamber by playing or fighting with other animals. Conditions were kept at 22–24 °C with a relative humidity of 50–60% using a 12-h day-night cycle. The animals had unrestricted access to standard pellet chow (Altromin, Lage, Germany) and tap water unless otherwise specified.

### 2.2. CR Regimen

**Figure 1:**
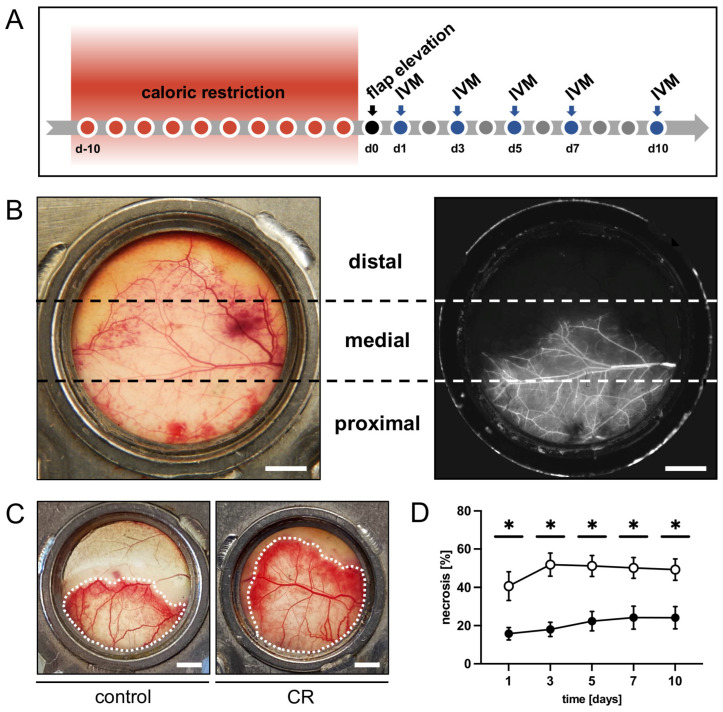
(**A**): Experimental protocol of the present study. Mice underwent a 10-day 30% caloric restriction (CR) before surgical flap elevation. Animals with unrestricted access to standard chow served as controls. Flap necrosis and vascularization were assessed by repeated intravital fluorescence microscopy (IVM) 1, 3, 5, 7 and 10 days after flap elevation. (**B**): Exemplary macroscopic image of an untreated control flap with the corresponding intravital fluorescent microscopic image on day 1 after flap elevation. Each flap was subdivided into three observational zones proximal, medial and distal to the flap’s base (marked by broken lines) for subsequent analyses. Scale bar: 2 mm. (**C**): Macroscopic images of the chamber observation window of a mouse from the untreated control group and a CR-treated mouse, showing a clear difference in vital flap tissue (indicated by dotted line). Scale bar: 2 mm. (**D**): Necrosis [%] of flaps in CR-treated mice (black circles, n = 8) and untreated controls (white circles, n = 8) 1, 3, 5, 7 and 10 days after flap elevation, as evaluated by intravital fluorescence microscopy and computer-assisted image analysis. Means  ±  SEM. * *p*  <  0.05 vs. control.

A total of 8 mice were randomly assigned to the CR group. After tracking their food intake to determine their average food consumption prior to the start of the experiments, the animals were transferred to a new cage to prevent them from consuming buried chow or mouse droppings and were provided with 70% of the previously consumed amount of chow. For a standard consumption of 5.1 ± 1.6 g/d of chow, they were provided with 3.6 g/d of chow. The standard consumption during the present study was similar to the amount referenced in other research [29]. Such a 30% CR has already been proven to be effective in previous studies [30,31,32]. The duration of the fasting intervention was based on similar interventions used in the past, spanning from 1 to 4 weeks of CR [11,33]. Moreover, a 10-day intervention would still be feasible in a clinical setting in human patients. After 10 days of 30% CR, a musculocutaneous flap was raised on the back of the animals (Figure 1A) and they had unrestricted access to food for the remaining observation period. Animals in the control group (n = 8) had unrestricted access to standard pellet chow (Altromin) throughout the entire experiment. 

### 2.3. Anesthesia

Both the surgical flap elevation and the dorsal skinfold chamber implantation were performed under general anesthesia. Narcosis was induced through intraperitoneal injection of ketamine (100 mg/kg body weight; Ursotamin^®^; Serumwerke Bernburg, Bernburg, Germany) and xylazine (12 mg/kg body weight; Rompun^®^; Bayer, Leverkusen, Germany). To prevent postoperative pain, all animals received a subcutaneous injection of buprenorphine hydrochloride (0.01 mg/kg body weight; Temgesic^®^; RB Pharmaceuticals Limited, Slough, UK). General anesthesia was induced in an analogue fashion to perform subsequent intravital fluorescence microscopy.

### 2.4. Flap Model

The flap model used herein has been described in detail in previous studies [34,35]. Briefly, musculocutaneous random pattern flaps were raised on the back of each animal. The flaps measured 15 mm (base) × 11 mm (length) and were positioned perpendicular to the spine. By elevating the flaps, the thoracodorsal artery and the deep circumflex iliac artery were severed, creating a random pattern flap. The chosen width-to-length-ratio induces ischemia of the distal portion of the flap. The tissue of untreated controls subsequently developed roughly 50% necrosis (Figure 1B). After flap elevation, the lateral wound margins were fixed back to the remaining dorsal skinfold and the flaps were finally sandwiched between two dorsal skinfold chamber frames (Irola Industriekomponenten GmbH and Co. KG, Schonach, Germany); this allowed direct access to the flap tissue through an observation window in one of the chamber frames that was sealed with a cover glass and a snap ring. The microcirculation within the flap tissue could be analyzed repeatedly over the course of a 10-day observation period by means of intravital fluorescence microscopy. As necrosis demarcates after 3–5 days, this observation period covers the crucial period during which ischemia-induced tissue damage occurs and has been well established in the past [34,35,36]. After the preparation, the animals were allowed to recover from the anesthesia and surgery for 24 h before the first microscopy was performed. Feeding and sleeping habits remained unchanged during the observation period, which is a sign that the animals were not subjected to undue stress.

### 2.5. Intravital Fluorescence Microscopy

Repeated intravital fluorescence microscopy was carried out on days 1, 3, 5, 7 and 10 after flap elevation. The blood plasma marker 5% fluorescein isothiocyanate (FITC)-labeled dextran (150,000 Da; Sigma-Aldrich, Taufkirchen, Germany) was used for contrast enhancement. It was applied to the anesthetized animals by means of retrobulbar intravenous injection. The animals were then fixed on a plexiglass platform to ensure the horizontal positioning of the chamber window, which was placed under a Zeiss Axiotech fluorescence epi-illumination microscope (Zeiss, Oberkochen, Germany). To record the flap microcirculation, a charge-coupled device video camera (FK6990; Pieper, Schwerte, Germany) and a DVD system were used. During each microscopy, a panoramic image of the chamber was recorded for planimetric measurement of the perfused tissue surface. For a detailed analysis of the tissue perfusion in the different regions of the flap, three observational zones—proximal, medial and distal to the flap base (Figure 1B)—were analyzed individually. For this purpose, two regions of interest (ROI) were chosen per zone containing an arterio-venous bundle. These bundles were recorded and could be identified by their distinct morphological aspects during each microscopy for repeated measurements. Two capillary fields adjacent to the arterio-venous bundles were recorded per ROI. Images of unperfused ROI were recorded if they could be safely identified. Individual ROI were lost to follow-up due to the deterioration of the tissue after the demarcation of necrosis. One additional ROI within the medial transition zone between the perfused and non-perfused tissue of each flap was documented with images and videos to assess possible new vessel formation. 

All imaging parameters were analyzed using the CapImage analysis system (Version 8.5, Zeintl, Heidelberg, Germany). Analysis was carried out offline after the microscopies. The rate of necrosis was calculated as 100 perfused surface area/total chamber surface area × 100) and given in %. The functional capillary density (FCD) was measured as the length of perfused capillaries per capillary field and expressed in cm/cm^2^. Within each ROI and the adjacent capillary fields, microhemodynamic parameters were measured in the arterioles, capillaries and venules. Vessel diameters (D) were measured perpendicular to the vessel path and expressed in µm. The centerline red blood cell (RBC) velocity (V) was determined using line shift diagrams [33]. V and D were used to calculate the volumetric blood flow (VQ) as VQ = π × (D/2)^2^ × V/K, where K (=1.6) represents the Baker-Wayland factor considering the parabolic velocity profile of blood in small vessels. VQ was expressed in pL/s. Neovessel formation within the transition zone was measured by quantifying the density of newly formed vessel sprouts and expressed in cm/cm^2^. In contrast to the capillaries of the panniculus carnosus muscle, which usually show a parallel arrangement, the newly formed vessel sprouts showed an irregular and entangled conformation and could thus be clearly distinguished [37].

### 2.6. Histology and Immunohistochemistry

Once the in vivo observation period was concluded, the flap tissue was harvested and fixed in formalin. The tissue samples were embedded in paraffin and sliced into 3-µm-thick sections. Hematoxylin and eosin (HE) staining was performed according to a standard protocol. Tissue analysis was performed using a BX60 microscope (Olympus, Hamburg, Germany) in combination with the cellSens Dimension 1.11 imaging software (Olympus). 

Immunohistochemical detection of myeloperoxidase-positive (MPO^+^) neutrophilic granulocytes and cleaved caspase (Casp)-3^+^ cells undergoing apoptosis was performed on individual sections. Antigens in the samples were demasked using citrate buffer. Subsequently, unspecific binding sites were blocked using goat serum. Cell staining was performed by incubating the samples with a polyclonal rabbit antibody against MPO (1:100; Abcam, Cambridge, UK) or a monoclonal rabbit antibody against Casp-3 (1:100; Cell signaling Technology, Danvers, MA, USA) as primary antibodies., A biotinylated goat anti-rabbit IgG antibody (ready-to-use; Abcam) was used as a secondary antibody. Peroxidase-labeled streptavidin (ready-to-use; Abcam) was then applied to detect the biotinylated antibody. Three-amino-9-ethylcarbazole (Abcam) was used as chromogen. Counterstaining of the tissue sections was performed using Mayer’s hemalum (Merck, Darmstadt, Germany). Two randomized high-power fields (HPFs) in the proximal and medial transition zones of the flaps were chosen for quantifying the detected cells. Distal necrotic flap tissue was excluded from the analysis. 

### 2.7. Statistical Analysis

All data were first tested for normal distribution and equal variance. Differences between the CR and control group were analyzed using an unpaired Student’s *t*-test (GraphPad Prism 9; GraphPad Software, San Diego, CA, USA). If data were non-parametric, a Mann-Whitney rank sum test was carried out. All values are expressed as means ± standard error of the mean (SEM). The threshold for statistical significance was set at a value of *p*  <  0.05.

## 3. Results

### 3.1. In Vivo Experiments

The intravital fluorescence microscopy revealed that over the course of the 10-day in vivo observation period, the flaps of the CR-treated animals exhibited a significantly lower rate of necrosis when compared to those of the untreated controls (Figure 1C,D). In both groups, the necrosis rate increased over time, as nutritive blood perfusion in some of the critically perfused distal flap zones could no longer be maintained (Figure 1D). This trend was most pronounced in the control animals between day 1 and 3. 

**Figure 2:**
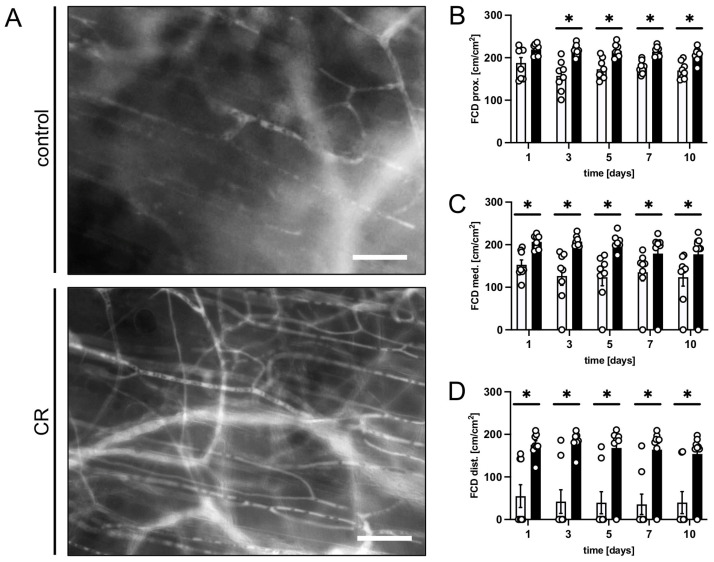
(**A**): Intravital fluorescent microscopic images of capillary beds in the distal flap tissue of a mouse from the untreated control group and a mouse from the CR-treated group on day 5 after flap elevation. Scale bar: 50 µm. (**B**–**D**): FCD [cm/cm^2^] as assessed in the proximal (**B**), medial (**C**) and distal zone (**D**) of flaps in CR-treated mice (black bars, n = 8) and untreated controls (white bars, n = 8) on days 1, 3, 5, 7 and 10 after surgical flap elevation, as detected by intravital fluorescence microscopy and computer-assisted image analysis. Means  ±  SEM. * *p*  <  0.05 vs. control.

All three observational zones of the CR-treated flaps exhibited a significantly higher FCD over the course of the experiments when compared to the controls (Figure 2A–D). The proximal and medial zones of the flaps in the CR-treated animals presented with an FCD of ~180–200 cm/cm^2^ (Figure 2B,C), while the distal zone still had a FCD of ~160 cm/cm^2^ (Figure 2D). The untreated animals showed markedly lower values in the proximal and medial zones (~120–170 cm/cm^2^) and even only ~40 cm/cm^2^ in the distal zone (Figure 2B–D).

In addition, we assessed the diameters and centerline RBC velocities in the arterioles, capillaries and venules of the flap tissue to calculate the volumetric blood flow. No marked differences in the volumetric blood flow in the arterioles, capillaries and venules of the flap were observed between the flaps in the untreated controls and the CR-treated animals (Table 1). However, the CR-treated animals had a tendency towards higher blood flow values when compared to the controls. A minor increase in blood flow could be detected in all vessel types of both groups over time as the flaps adjusted to the changed perfusion. 

**Figure 3:**
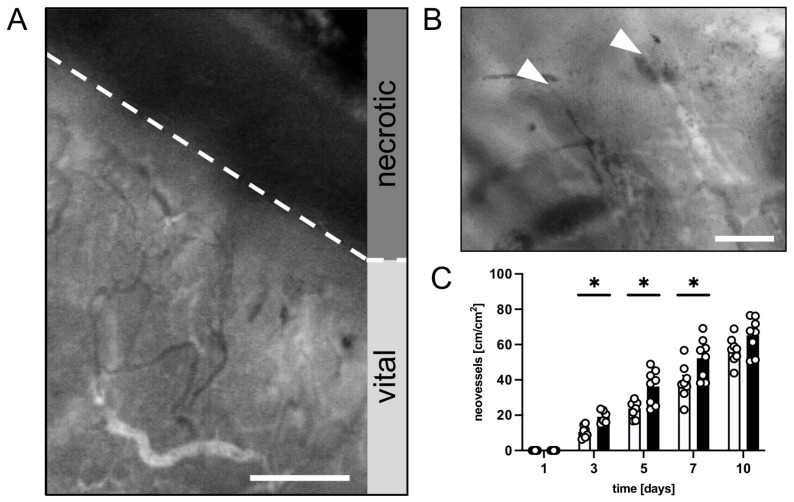
(**A**): Intravital fluorescent microscopic image of the transition zone between vital and necrotic tissue (border indicated by broken line) of a flap in a CR-treated mouse on day 10 after flap elevation. Scale bar: 200 µm. (**B**): Intravital fluorescent microscopic image of angiogenic vessel sprouts (marked by white arrowheads) in the transition zone of a flap in a mouse from the CR-treated group on day 10 after flap elevation. Scale bar: 50 µm. (**C**): Neovessels [cm/cm^2^] in the medial transition zone of flaps in CR-treated animals (black bars, n = 8) and untreated controls (white bars, n = 8) on days 1, 3, 5, 7 and 10 after surgical flap elevation, as assessed by intravital fluorescence microscopy and computer-assisted image analysis. Means  ±  SEM. * *p*  <  0.05 vs. control.

Finally, new vessel formation was assessed in the transition zone between the vital and necrotic flap tissue. Both groups showed changes in the capillary architecture starting on day 3 after the surgical flap elevation. Angiogenic sprouts grew out of the parallelly arranged capillaries of the panniculus carnosus muscle, which had dilated and become irregular in shape and diameter (Figure 3A,B). The CR-treated animals showed a faster onset of angiogenesis with significantly more newly formed microvessels between days 3 and 7 when compared to the untreated controls (Figure 3C).

### 3.2. Histological and Immunohistochemical Analysis

Additional histological and immunohistochemical analyses were performed after the in vivo experiments. The HE-stained sections were used for the initial assessment of the tissue morphology and the identification of the transition zone between the vital and necrotic flap tissue in the medial observational zone. The distal zone was excluded from further immunohistochemical analyses due to tissue necrosis.

**Figure 4:**
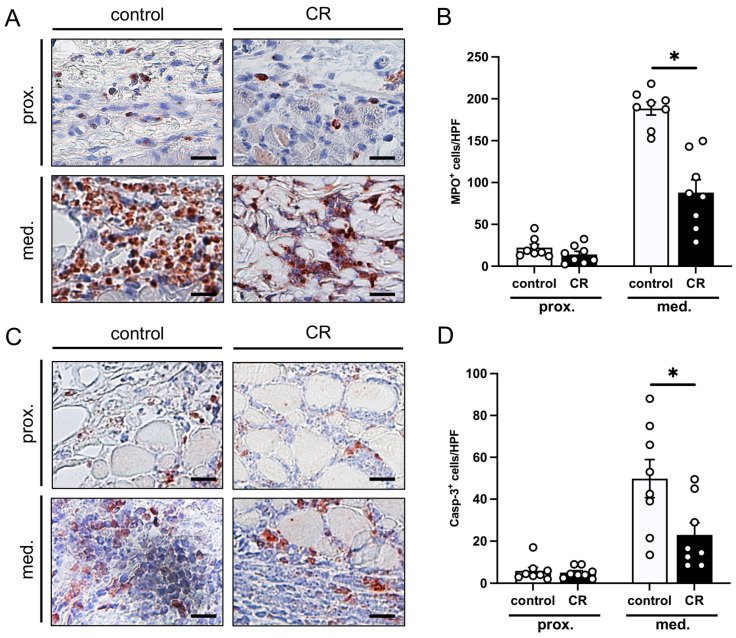
(**A**): Microscopic images of immunohistochemically stained sections of the proximal and medial zone of flaps in a mouse from the untreated control group and a mouse from the CR-treated group on day 10 after flap elevation. An antibody against the neutrophilic granulocyte marker MPO was used for immunohistochemical staining. Scale bar: 20 µm. (**B**): MPO^+^ cells/HPF in the proximal and medial zones of flaps in CR-treated mice (black bars, n = 8) and untreated controls (white bars, n = 8) on day 10 after flap elevation, as assessed by immunohistochemistry. (**C**): Microscopic images of immunohistochemically stained sections of the proximal and medial zone of flaps in a mouse from the untreated control group and a mouse from the CR-treated group on day 10 after flap elevation. An antibody against the apoptotic marker Casp-3 was used for immunohistochemical staining. Scale bar: 20 µm. (**D**): Casp-3^+^ cells/HPF in the proximal and medial zones of flaps in CR-treated mice (black bars, n = 8) and untreated controls (white bars, n = 8) on day 10 after flap elevation, as assessed by immunohistochemistry. Means  ±  SEM. * *p*  <  0.05 vs. control.

The immunohistochemical detection of MPO showed significantly more invading neutrophilic granulocytes in the medial transition zone of the flaps in the control animals (Figure 4A,B). The CR-treated animals displayed a suppressed inflammatory reaction in this zone, as evidenced by a lower number of MPO^+^ cells per HPF when compared to the untreated control mice (Figure 4B). In the proximal flaps’ base, there was no significant difference between the number of MPO^+^ cells per HPF. 

The detection of Casp-3^+^ cells revealed low numbers of apoptotic cells in the proximal zone of the flaps in both groups (Figure 4C,D). In contrast, the medial transition zone showed a markedly higher number of cells undergoing apoptosis. Notably, this number was significantly lower in the flaps of the CR-treated animals when compared to the untreated controls (Figure 4D).

## 4. Discussion

CR improves overall health of organisms by means of several effects acting in a synergistic manner. On a cellular level, various contributing mechanisms have been proposed, including reduced fasting insulin levels, lower resting energy expenditure and suppressed oxidative stress within the tissue [2,38]. Some of these effects have been shown to be highly beneficial in the context of surgical interventions in general, and in flap surgery in particular [39,40]. Hence, it is tempting to speculate that the improved cellular functionality and integrity results in increased ischemic tolerance and may promote flap survival under ischemic tissue stress. Although DR was initially studied to promote healthy aging and lifespan extension, preventive dietary recommendations have been proposed for the protection from the acute stress of a surgical intervention in the past. Proof-of-concept studies dating back to the 1990s have been performed in several preclinical rat models [12,41,42]. More recent studies have confirmed the effects of preoperative DR against ischemic damage in the liver, kidney and brain [13,15,33].

Based on this promising research, we have investigated the possible tissue-protective effects of CR in critically perfused musculocutaneous flaps. We found that the rate of tissue necrosis is significantly reduced by a 10-day—and therefore relatively short-term—CR pretreatment before flap elevation using a modified murine dorsal skinfold chamber model. This beneficial effect is mediated by maintaining nutritive tissue perfusion and suppressing ischemia-induced inflammation and apoptosis.

It should be noted that the above-mentioned effects were examined in a relatively small number of animals and with a limited 10-day follow up period for the present study. Moreover, no assessment of the metabolic outcomes, inflammatory mediators or angiogenic factors was performed to shed more light on the mechanisms involved, and these factors should be included in further research. 

The beneficial effects of CR on ischemic tissue are multifaceted. By shifting the cells towards survival programs and slowing their metabolism, they become more resistant to ischemic stress under fasting conditions [43]. This effect could also be observed in the present study, in which significantly less Casp-3^+^ cells undergoing apoptosis in the transition zone of the flaps in the CR-treated animals were detected. Moreover, DR upregulates the expression of stress response proteins, such as hypoxia inducible factor (HIF)-1α, which also mediate angiogenesis [44,45]. CR has further been shown to stimulate revascularization in a murine ischemic hindlimb model via an adiponectin-mediated activation of endothelial nitric-oxide synthase (eNOS) [46]. An increased angiogenic response to the ischemic stimulus of flap elevation could also be detected in the present study. In addition, the CR-treated animals showed a tendency toward higher blood flow values in the arteries, capillaries and venules when compared to the untreated controls, although the differences were not statistically significant. CR has also been shown to reduce oxidative stress and inflammation [47]. In line with previous studies, a markedly lower rate of invading neutrophilic granulocytes was detected in the transition zone of the flaps in the fasting animals. Dampening the inflammatory reaction is highly beneficial as invading immune cells cause parenchymal cell dysfunction and influence blood rheology in a negative fashion [48,49].

Recent findings indicate that the positive effects of CR are also mediated by shifts in the gut microbiome [50,51,52]; it acts as a key regulator of nutrient bioavailability and intestinal physiology. By changing the composition of the gut microbiome, DR positively influences regulatory T cell differentiation and the body’s cytokine profile [53,54]. Anderson et al. [55] were able to show that a fasting period as short as a 4-day 30% CR causes distinct alterations to the microbiome composition. In fact, they found protein and caloric restriction to cause an increase in several short chain fatty acid (SCFA)-producing bacteria. Among them were the primary producers of butyrate, which acts as an anti-inflammatory through the suppression of the NF-kB pathway and the differentiation of regulatory T cells [51,52,56]. Of interest, effects such as the upregulation of butyric acid-producing species in the gut microbiome after DR have also been demonstrated in humans [57]. 

The concept of using DR to counteract surgical stress is particularly appealing as the positive effects of DR have already been studied in humans. For instance, several studies have been conducted to test the safety and feasibility of a DR intervention before vascular surgery [24,58,59]. Similarly, Grundmann et al. [28] were able to show a reduced incidence of acute kidney injury after cardiac surgery following a 40% reduction in caloric intake for the duration of 7 days before surgery. The feasibility of preoperative CR has also been tested in morbidly obese patients without any major side effects [60]. Based on these promising findings, the translation of CR conditioning into clinical routines for other indications, such as flap surgery, would likely be similarly feasible. Of note, the anti-inflammatory effect of DR has also previously been demonstrated in humans. Using the large and readily available pool of fasting individuals during the Muslim religious fast of Ramadan, researchers showed an immunomodulatory effect with a reduced secretion of pro-inflammatory cytokines, such as interleukin (IL)-1β, IL-6 and tumor necrosis factor (TNF)-α, after fasting [61,62].

## 5. Conclusions

Taken together, the present preclinical study demonstrates that a preoperative CR of 30% during a 10-day period effectively decreases the necrosis of musculocutaneous random pattern flaps in mice. The reduced caloric intake maintained nutritive perfusion and reduced inflammation and apoptotic cell death within the flap tissue. These promising findings suggest that CR is a promising novel conditioning approach to improving the survival of surgical flaps. Hence, large-scale clinical trials are now required to also prove the effectiveness of preoperative CR in humans. Moreover, future research should assess the metabolic outcomes and cytokine profiles in order to shed more light on the mechanisms involved.

## Figures and Tables

**Table 1:** Volumetric blood flow [pL/s] of arterioles, capillaries and venules in the proximal, medial and distal zone of flaps in untreated control mice (n = 8) and mice subjected to a 30% caloric restriction (CR; n = 8) 1, 3, 5, 7 and 10 days after flap elevation, as assessed by intravital fluorescence microscopy and computer-assisted image analysis.

Volumetric Blood Flow [pL/s]		d1	d3	d5	d7	d10
Arterioles						
prox.	control	474 ± 67	699 ± 147	848 ± 143	974 ± 173	1363 ± 248
	CR	791 ± 113 *	931 ± 159	1089 ± 164	1626 ± 235 *	1586 ± 211
med.	control	375 ± 109	502 ± 115	556 ± 93	822 ± 193	1060 ± 211
	CR	573 ± 109	813 ± 138	871 ± 139	1140 ± 174	1160 ± 195
dist.	control	208 ± 42	414 ± 120	681 ± 135	480 ± 205	867 ± 446
	CR	377 ± 66	524 ± 84	821 ± 167	899 ± 98	1125 ± 161
Capillaries						
prox.	control	1 ± 0	2 ± 0	3 ± 1	5 ± 1	7 ± 1
	CR	2 ± 0 *	3 ± 0 *	5 ± 0	8 ± 1 *	11 ± 1
med.	control	2 ± 0	2 ± 0	3 ± 0	5 ± 1	6 ± 1
	CR	2 ± 0	3 ± 0 *	5 ± 0 *	7 ± 1	9 ± 1
dist.	control	1 ± 0	2 ± 0	3 ± 1	6 ± 1	6 ± 1
	CR	2 ± 0	3 ± 0	4 ± 1	7 ± 1	10 ± 2
Venules						
prox.	control	595 ± 146	921 ± 188	1459 ± 272	2048 ± 438	2440 ± 416
	CR	1538 ± 332 *	1800 ± 374	2199 ± 456	3470 ± 801	3789 ± 926
med.	control	607 ± 159	1208 ± 519	1453 ± 283	1934 ± 443	2469 ± 413
	CR	1116 ± 277	1431 ± 326	1905 ± 337	2629 ± 754	3240 ± 796
dist.	control	125 ± 47	589 ± 119	762 ± 328	1508 ± 479	1522 ± 93
	CR	361 ± 86	703 ± 214	1272 ± 301	1505 ± 127	1903 ± 362

Mean ± SEM. * *p* < 0.05 vs. control.

## Data Availability

All data can be obtained in this manuscript.
